# Successful resuscitation of a patient with pernicious placenta previa and placenta accreta who had massive life-threatening bleeding during cesarean section: A case report

**DOI:** 10.1097/MD.0000000000015025

**Published:** 2019-04-05

**Authors:** Xiaoqin Jiang, Xuemei Lin, Xueguang Han, Yushan Ma, Fumin Zhao

**Affiliations:** a Anesthesiology Department of West China Second University Hospital, Sichuan University; b Key Laboratory of Birth Defects and Related Diseases of Women and Children (Sichuan University), Ministry of Education; c Radiology Department of West China Second University Hospital, Sichuan University, Sichuan Province, China.

**Keywords:** case report, hysterectomy, life-threatening bleeding, pernicious placenta previa

## Abstract

**Rationale:**

Pernicious placenta accrete (PPP) is an obstetrical complication associated with severe life-threatening hemorrhage, which is one of the leading causes of maternal mortality worldwide. Caesarean hysterectomy is the effective method to control intraoperative bleeding for this unscheduled high-risk patient. But a challenge for clinicians in this case is to determine the optimal timing of hysterectomy, because it will directly determine maternal outcome.

**Patient concerns:**

We here report a case diagnosed with PPP who suffered from a severe life-threatening hemorrhage during cesarean section but was successfully resuscitated and subsequently discharged from hospital after a smooth recovery.

**Diagnoses:**

Although binding the lower uterine segment with a tourniquet markedly reduced bleeding in the surgical field after delivery, massive concealed vaginal life-threatening bleeding occurred immediately, and the amount of vaginal blood loss within 10 minutes was as much as 3000 mL.

**Interventions:**

An experienced multidisciplinary team was immediately established, and an unscheduled caesarean hysterectomy was performed immediately, and cell salvage was used.

**Outcome:**

The patient was successfully resuscitated and both the parturient and neonate were well and discharged.

**Lesson:**

If binding the lower uterine segment with a tourniquet markedly reduces bleeding in the surgical field after cesarean delivery in high-risk patients with PPP, and persistence of hypotension after active resuscitation of the circulation is detected, anesthesiologist should be vigilant enough to detect the possibility of concealed vaginal life-threatening bleeding. If this is confirmed, it should be quickly identified whether bleeding can be quickly controlled within a short period of time. If not, the preferred strategy is that the earlier the unscheduled hysterectomy, the better the outcome. A well-established multidisciplinary team and autologous blood recovery and transfusion techniques are also important in ensuring successful resuscitation of patients.

## 1 Introduction

Pernicious placenta accrete (PPP) is an obstetrical complication associated with severe life-threatening hemorrhage^[1–3]^ which is consistently a leading cause of maternal morbidity and mortality worldwide,^[4,5]^ accounting for over 27% of maternal deaths. Importantly, obstetric hemorrhage is one of the more treatable causes of maternal mortality, and demonstrating improvement in outcomes has been challenging. Caesarean hysterectomy is the effective method to control intraoperative bleeding for this unscheduled high-risk patient. But a challenge for clinicians in this case is to determine the optimal timing of hysterectomy, because it will directly determine maternal outcome. We here report a case with PPP and placenta accreta who had a massive concealed vaginal life-threatening hemorrhage during cesarean section (CS) but was successfully resuscitated and subsequently discharged from hospital after a smooth recovery.

## 2 Case report

A 38-year-old woman (gravida 5, para 3^+1^) had been delivered of 2 healthy sons and 1 healthy daughter via lower segment CSs in other hospitals 15, 11, and 8 years, respectively, prior to the current presentation. At 32^+1^ weeks of gestation, she presented at the emergency department of our hospital because of spotting. Her current pregnancy was not registered with any hospital and she had received no antenatal care. She had noted intermittent minor spotting between 28 and 30 weeks of gestation but had not visited any hospital or received any treatment.

**Figure 1:**
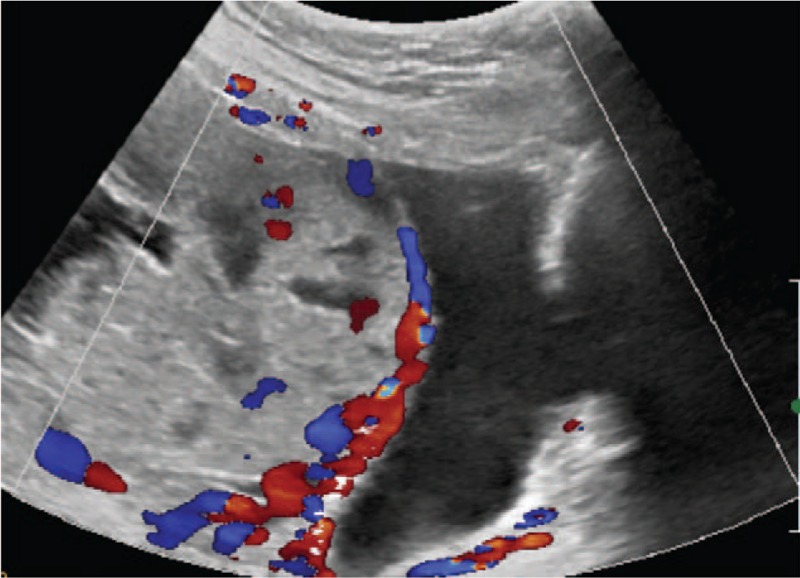
Ultrasound images before cesarean section. It showed placenta, fluid dark areas in placenta, and rich blood flow between placenta and anterior wall of lower segment of uterine.

**Figure 2:**
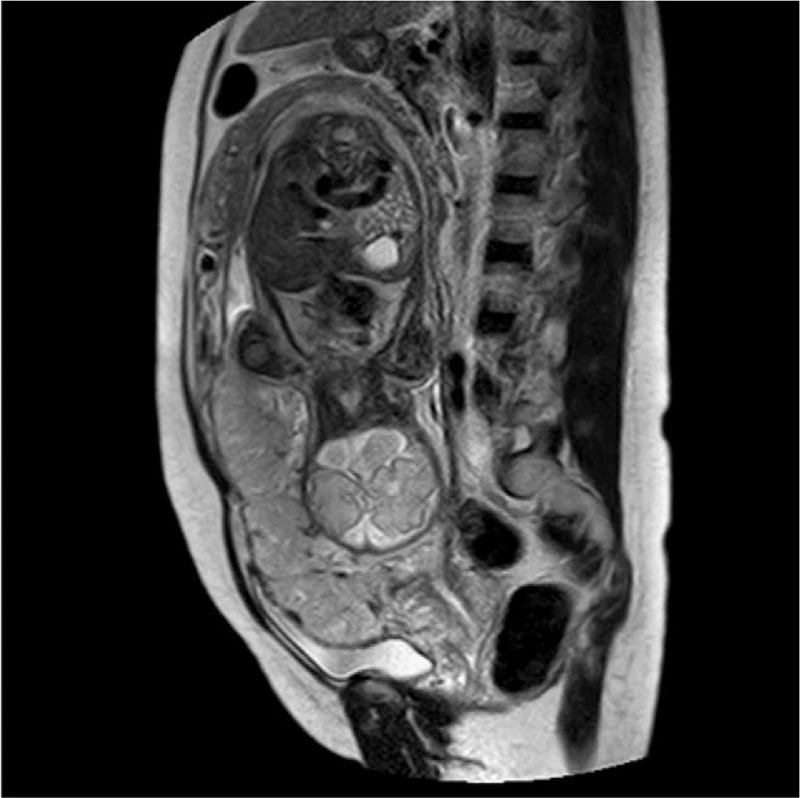
Mangnetic resonance imaging revealed placenta completely covered the internal cervical orifice, and diffuse placenta accreta and placenta percreta on the lower anterior uterine wall were visible.

On presentation to our hospital, she was found to have a hemoglobin (HGB) concentration of 87 g/L. Ultrasonography revealed that the lower edge of the placenta was completely covering the internal cervical orifice and was suspicious of the presence of PPP (Fig. 1). A plain magnetic resonance imaging (MRI) revealed that the pernicious placenta previa was completely covering the internal cervical orifice, she had diffuse placenta accreta and also placenta percreta on the lower anterior uterine wall, and distended and twisted vessels were visible on the surface of the lower anterior uterine wall (Fig. 2). A diagnosis of PPP was made. The patient was admitted to the obstetric ward and treated with dexamethasone to promote pulmonary maturation.

At 33 weeks gestation, she had 450 mL vaginal bleeding and was immediately transferred to the operation room for emergency CS. On arrival at the operating room, her blood pressure was 125/75 mm Hg and heart rate 90 beats/min. A resuscitation team comprising senior staff of the departments of gynecology, obstetrics, anesthesiology, and neonatology, the intensive care unit (ICU), blood bank and laboratories, and a nursing team was immediately established. The anesthesiologist immediately punctured an artery to enable invasive blood pressure (BP) monitoring, and devices for autologous blood recovery were assembled. Nurses established four 16 G intravenous lines immediately for fluid infusion. The cumulative preoperative blood loss was 1290 mL (including the 450 mL episode). Because the patient's preoperative HGB was 87 g/L and she had known placenta accreta, 4.5 IU of blood were obtained from the blood bank before CS.

**Figure 3:**
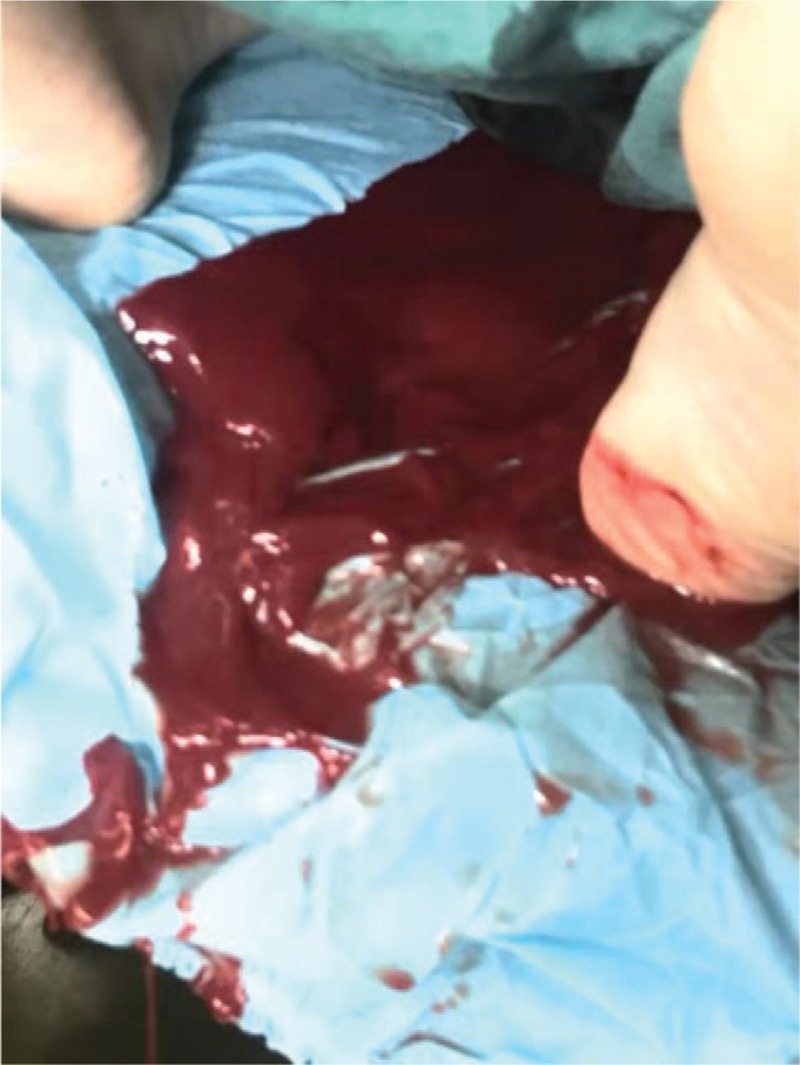
The picture shows a large amount of blood had flowed from the vagina onto and beneath the operating table as a result of the failure of the cervix to contract.

Remifentanil 70 μg, propofol 120 mg, and succinylcholine 100 mg were intravenously infused, and tracheal intubation performed under general anesthesia. The infant was delivered 4 minutes after commencing the operation, had an Apgar score of 8-9-9, and was transferred to the neonatal ICU for further treatment. Rapid and massive bleeding occurred immediately after delivery but was decreased markedly in the surgical field by binding the lower uterine segment with a tourniquet. However, the BP inexplicably remained at about 75/45 mm Hg despite rapid blood and fluid transfusion. The explanation for this became evident when it was found that a large amount of blood had flowed from the vagina onto and beneath the operating table as a result of the failure of the cervix to contract (Fig. 3). An emergency protocol for massive bleeding was immediately initiated. After an on-the-table consultation, hysterectomy was performed immediately.

The total intraoperative blood loss was 13,400 mL. The patient was transfused with autologous blood (1781 mL), allogeneic leukocyte-free red blood cell suspension (21 IU), fresh frozen plasma (2200 mL), cryoprecipitate (6 IU), machine-collected platelets (1 IU), fibrinogen (4 g), crystalloid fluid (8700 mL), and hydroxyethyl starch (1000 mL). Upon completion of the surgery, her HGB was 76 g/L and activated partial thromboplastin time (APTT) 48 seconds.

**Figure 4:**
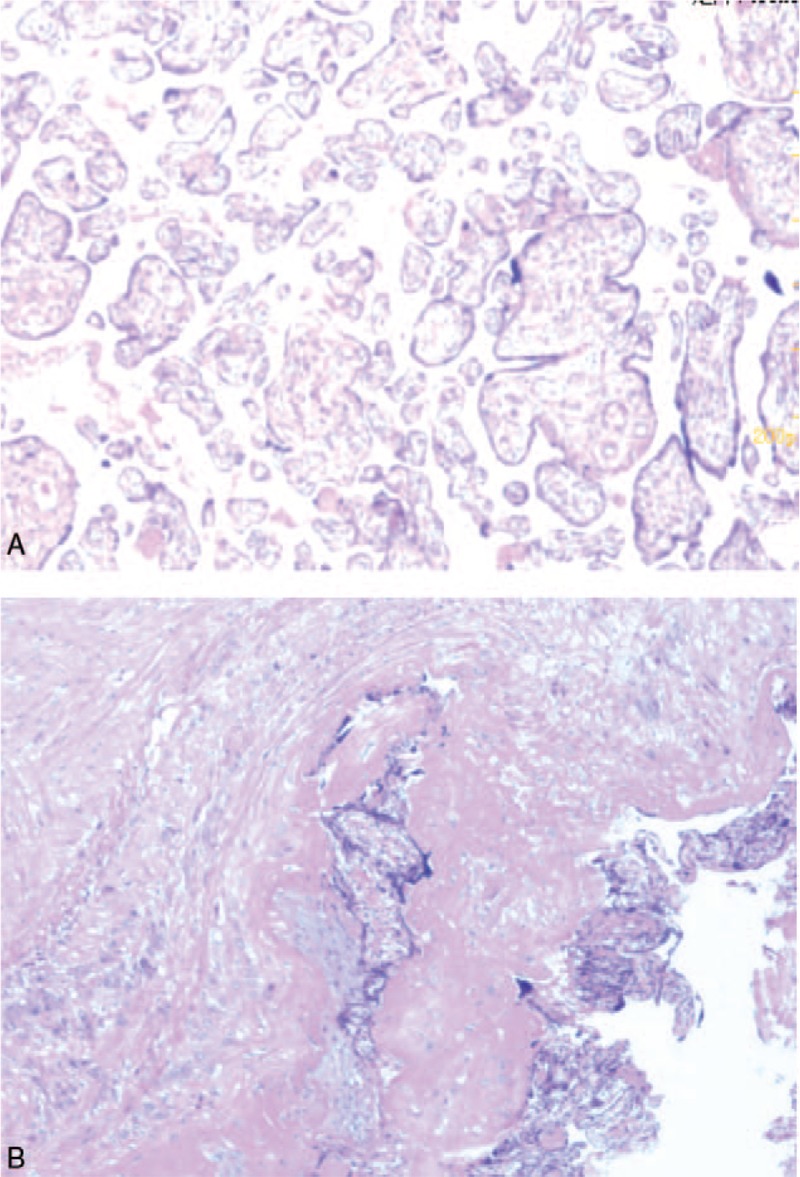
Postoperative pathologic examination of the operative specimen confirmed the presence of placenta accreta.

Postoperative inspection of the uterus confirmed placenta accreta in the anterior uterine wall of the lower uterine segment. Parts of the placenta had implanted into the cervical canal, particularly its posterior wall, where it almost reached the external cervical orifice. Postoperative pathologic examination of the operative specimen again confirmed the presence of placenta accreta (Fig. 4). Postoperatively, the patient was returned to the ward safely and recovered well. Her HGB was 71 g/L on the 2nd postoperative day. On the 6th postoperative day, her HGB was 81 g/L and the APTT and prothrombin time were normal. Both the parturient and neonate were well and discharged.

## 3 Discussion

Potentially fatal massive bleeding often occurs during CS in patients with PPP, especially when accompanied by placenta accreta. According to published reports, the average blood loss during CS in patients with PPP accompanied ranges from 3000 to 5000 mL,^[6]^ exceeding 10,000 mL in 10% of such patients. Massive bleeding can cause dysfunction and failure of the heart, lungs, brain, liver, kidneys, and other vital organs and is potentially fatal.

The patient had undergone 3 CSs prior to the current pregnancy and was thus at high risk of placenta accreta in this, her 4th CS. Preoperative ultrasound/placental MRI and postoperative examination of the uterus identified and confirmed placenta accreta. Parts of the placenta had implanted into the cervical canal, even reaching the external cervical orifice. Therefore, when binding the lower uterine segment with a tourniquet markedly reduce bleeding in the surgical field after delivery, massive concealed vaginal life-threatening bleeding occurred immediately because the lower uterus segment did not contract, and the amount of vaginal blood loss within 10 minutes was as much as 3000 mL, total blood loss being 13,400 mL during the whole operative procedure. An experienced multidisciplinary team was immediately established, and a total hysterectomy was performed immediately, and cell salvage was used. The patient was successfully resuscitated and recovered well postoperatively.

Effective methods to control intraoperative bleeding are important for this patient because they will directly determine maternal outcomes. What we learnt from our experience in resuscitating this patient with PPP can be summarized as follows.

First, in women strongly suspected of having PPP, a tourniquet can be used to bind the lower uterine segment to reduce bleeding after CS. In the current patient, binding of the lower uterine segment markedly reduced bleeding in the surgical field. However, she remained hypotensive despite balanced input and output of fluids according to estimated blood loss in the surgical field. The explanation for this became evident when it was found that a large amount of blood had flowed from the vagina onto and beneath the operating table as a result of the failure of the cervix to contract. Subsequent weighing of the blood on and beneath the operating table revealed that she had lost about 3000 mL via this route within the first 10 minutes after delivery. Therefore, in women at high risk of placenta accreta extending into the internal cervical orifice, the risk of massive concealed transvaginal life-threatening bleeding should be considered during CS. It is very important, because in this life-threatening emergency (3000 mL per 10 minutes), the earlier the hysterectomy, the better the outcome.

Second, an experienced multidisciplinary rescue team was essential for the successful management of this patient. When sudden, heavy (450 mL) vaginal bleeding occurred, a resuscitation team comprising senior staff from the departments of gynecology, obstetrics, anesthesiology, and neonatology, the ICU, blood bank, and laboratories, and a nursing team was immediately established. An emergency protocol for massive bleeding was immediately initiated when massive concealed transvaginal life-threatening bleeding was detected. The gynecologist removed the uterus in a timely and skillful manner; the anesthesiologist cooperated efficiently with the nursing team in blood transfusion, rehydration, and correcting coagulation disorders; blood bank staff ensured timely supply and delivery of allogeneic blood and coagulation factors to the anesthesiologist. Therefore, an experienced multidisciplinary team is strongly recommended for minimizing maternal mortality and improving outcomes of such high-risk patients undergoing cesarean resection.

A prophylactic abdominal aorta balloon catheter is often inserted preoperatively^[7,8]^ to help control intraoperative bleeding in women with PPP by occluding the abdominal aorta as necessary, thus contributing to maintaining hemodynamic stability and gaining time for resuscitation. However, our patient's severe bleeding was sudden and unexpected, leaving insufficient time for any preoperative interventions. The most important goal was to control bleeding, the ability to do this determining whether the uterus can be preserved. The timing of total hysterectomy is crucial to the prognosis of high-risk patients who have not had an occluding balloon placed in the abdominal aorta preoperatively. After using a tourniquet to ligate the lower uterine segment, our patient still lost a large amount of blood via the vagina because the cervix did not contract. The gynecologist therefore made an on-the-spot decision to quickly remove the uterus, thus minimizing further blood loss and increasing the chance of successful resuscitation by the resuscitation team.

Shed blood from the surgical field was collected, and a total of 1781 mL of autologous red cell washed using intravenous saline 0.9% was re-infused back into our patient during the operation, and it was a key part of her successful resuscitation. During autologous red cell re-infusion, a leukocyte filter was used to minimize the risk of amniotic fluid embolism. The large amount of blood lost via the vagina flowed onto and beneath the operating table and therefore could not be collected, necessitating supplementation by allogeneic blood transfusion. The application of autologous blood re-transfusion technology^[9,10]^ in obstetrics has been reported by a number of authors. In 2013, the UK guidelines on autologous blood transfusion listed obstetric surgery as one of the indications for this procedure for the 1st time; in the 2015, guidelines for obstetric anesthesia also recommended implementing autologous blood recovery techniques if the estimated blood loss exceeds 1500 mL. As demonstrated by our patient, salvaged autotransfusion resulted in no severe complications such as amniotic fluid embolism and was thus a practical and effective means of conserving allogeneic blood.

In summary, in high-risk patients with PPP, if binding the lower uterine segment with a tourniquet markedly reduces bleeding in the surgical field after cesarean delivery, and persistence of hypotension after active resuscitation of the circulation is detected, anesthesiologist should be vigilant enough to detect the possibility of concealed vaginal life-threatening bleeding. If it is confirmed, it should be quickly identified whether bleeding can be quickly controlled within a short period of time. If not, the earlier the hysterectomy, the better the outcome. A well-established multidisciplinary team and autologous blood recovery and transfusion techniques are also important in ensuring successful resuscitation of patients.

## Author contributions

**Data curation:** Xueguang Han.

**Project administration:** Xuemei Lin.

**Resources:** Fumin Zhao.

**Writing – Original Draft:** Xiaoqin Jiang.

**Writing – Review & Editing:** Yushan Ma.
